# Neurological manifestations of mycetoma: a cross-sectional community-based study

**DOI:** 10.1097/MS9.0000000000000122

**Published:** 2023-03-15

**Authors:** Elkhansa A. Ali, Khabab Abbasher Hussien Mohamed Ahmed, Radi Tofaha Alhusseini, Abdallah M. Abdallah, Muaz A. Ibrahim, Amira Siddig, Eilaf O. Bahi, Mohammed Mahmmoud Fadelallah Eljack, Abbasher Hussien

**Affiliations:** a Faculty of Medicine, University of Khartoum; b Faculty of Medicine, Alzaiem Alazhari University; c Faculty of Medicine, University of Bahri; d Faculty of Medicine, Al Neelain University, Khartoum; e Faculty of Medicine, University of Shendi, Shendi, Sudan; f Faculty of Medicine and Health Sciences, University of Bakht Alruda, Ad Duwaym, Sudan

**Keywords:** mycetoma, neurology, tropical diseases, tropical medicine

## Abstract

Mycetoma is a chronic specific granulomatous progressive and disfiguring subcutaneous inflammatory disease. It is caused by true fungi (Eumycetoma) or by higher bacteria (actinomycetoma). Mycetoma mainly affects the lower limbs, followed by the upper limbs, back, and rarely the head and neck. Mycetoma is mainly transmitted through trauma with infected sharp objects. Herein, we want to determine the neurological manifestations of mycetoma in Sudanese patients.

**Methodology:**

A descriptive cross-sectional community-based study included 160 patients with mycetoma seen in the White Nile state. A team of doctors collected data using standardized questionnaires that included clinical history, neurological examination, investigations including laboratory, neurophysiological studies, and imaging.

**Results:**

Almost 160 patients were included in the study; 90% of them were male. Two patients presented with entrapment neuropathy, one presented with proximal neuropathy, one had peripheral neuropathy, one had dorsal spine involvement and presented with spastic paraplegia with sensory level, one had cervical cord compression, and one patient had repeated attacks of convulsion.

**Conclusion:**

Although it is rare, clinicians should highly suspect neurological involvement in mycetoma patients.

Highlights

- Mycetoma is one of the most overlooked health problems in the world.
- Neurological manifestations of mycetoma are very rare.
- In Sudan, 49 cases of head and neck, mycetoma were reported by the Mycetoma Research Center.
- Mycetoma infection (whether bacterial or fungal) can cause peripheral or central nervous system damage.

## Introduction

Carter first recorded mycetoma, also known as Madura’s foot, in 1861. It is one of the most overlooked health problems in the world. It is commonly seen in the tropical and subtropical regions and usually occurs within 15 south to 30 north of the equator^1^,^2^. Mycetoma is a chronic specific granulomatous, progressive, and disfiguring subcutaneous inflammatory disease. It is caused by true fungi or higher bacteria, so it is usually classified as true fungi and actinomycetes, respectively^2^. The most common causative agents include the fungus *Madurella mycetomatis* and the actinomycetes *Nocardia brasiliensis*, *Actinomadura madyrae*, *Streptomyces somaliensis*, and *Actinomadura pelletier*. Male predominance is a constant finding in mycetoma. No age is exempted, but it commonly affects adults between 20 and 40 years of age, who represent the earning members of society, especially in underdeveloped countries. The organisms are usually present in the soil in the form of grains. This infecting agent is then implanted into the host tissue through a breach in the skin reduced by trauma caused by sharp objects example, a thorn in areas where mycetoma is frequent, the habit of going barefoot is common^3^. The trial of painless subcutaneous mass, sinuses formation and purulent and seropurulent discharge that contain grains is pathognomonic of mycetoma. It may spread to involve the skin and deep structures like nerves and bones, resulting in destruction, deformity, and loss of functions^4^,^5^.

Neurological manifestations of mycetoma are very rare. Known neurological manifestations of mycetoma include spinal cord myelopathies, and brain space-occupying lesions, rarely, mycetoma can present with neuropathy, myositis, proximal myopathy, and cranial nerve involvement. Most of the research on the neurological manifestations of mycetoma were case reports or case series. Mycetoma of the spine leads to the destruction of vertebrae and compression of the spinal cord, which eventually can lead to quadriplegia or paraplegia^4^,^6^. Head and neck mycetoma is a rare, severe, and debilitating disease with a low cure rate and high drop-out percentage^7^–^9^. In Sudan, 49 cases of head and neck, mycetoma were reported by the Mycetoma Research Center during the period 1991–2014. The majority were caused by actinomycete. The most common presentation was headache, followed by seizure and hemiplegia. Computed tomography scan revealed osteosclerotic rather than osteolytic lesions.

Concerning the neurological manifestations of mycetoma, several problems arise, including mycetoma space-occupying lesions, and myelopathy is the only investigated manifestation until now, while other neurological manifestations like peripheral neuropathy, proximal myopathy, and cranial nerves involvement were not investigated. This problematic scene points to the importance of conducting a cross-sectional study to determine the different varieties of neurological manifestations of mycetoma. Herein, we aimed to investigate the presence of neurological manifestations in Sudanese mycetoma patients.

## Materials and methods

### Study design

A descriptive cross-sectional community-based study was conducted in Al-Andalus village, White Nile State, about 250 km to the south of Khartoum. About 160 patients diagnosed with mycetoma were included in the study. Those who refused to participate were excluded from the study.

### Data collection methods

Data were collected using standardized questionnaires including clinical history, neurological examination, and investigations (urine, complete hemogram, blood urea nitrogen, serum creatinine, spinal MRI, brain MRI, creatinine phosphokinase, muscle biopsy, nerve conduction study, and electromyography).

Data management and analysis: By using SPSS (Statistical Package for the Social Sciences) software (version 20).

This study followed STROCSS (Strengthening The Reporting Of Cohort Studies in Surgery) 2021 checklist for cross-sectional studies^10^.

## Results

Out of 160 patients with mycetoma, 95% were male and 5% were female. The age distribution of our studied group ranges from 18 years to 78 years (80% of our patients are between 20 and 40 years). All of our patients are farmers. All of our patients are of low socioeconomic status and health education level. Lower limbs were affected in 80% of our patients, followed by upper limbs in 15%, while the trunk, back, neck, and head constituted 5%. Almost 95% of the patients had painless lesions.70% of our patients had a history of discharge containing grains, and the color of the grains was mainly yellow. One patient presented with entrapment neuropathy, one presented with proximal neuropathy, one patient had peripheral neuropathy, one patient had dorsal spine involvement and presented with spastic paraplegia with sensory level, one of our patients had cervical cord compression, while one patient repeated attacks of convulsion due to tumor-like mass caused by a fungal infection affecting the right cerebral hemisphere.

## Discussion

Like what was mentioned in the literature, our study showed that males were affected more than females because mycetoma infection commonly affects farmers, field laborers, and herdsmen whose occupation involves direct contact with the soil^6^. Although no age is exempted from the infection with mycetoma, the majority of our patients range from 18 to 45; this is similar to what was reported by other researchers worldwide^6^. The foot was found to be the most predominantly affected part of the body, followed by the hand and rarely other parts of the body like the thigh, trunk, back, neck, and head^7^. A considerable number of our patients (90%) had an obvious history of local trauma. The characteristic triad of a progressive, painless subcutaneous swelling at the site of previous trauma as well as the nodules, may suppurate and drain through multiple sinus tracts, and these sinuses may close transiently after discharge during the active phase of the disease. Fresh adjacent sinuses may open while they are connected, with deep sterile abscesses and with skin surfaces. Mycetoma is usually painless; it was suggested that mycetoma produces substances that have anesthetic action^8^. At a late stage of the disease, the pain may become negligible due to nerve damage by the tense fibrous tissue reactions, endarteritis obliterans, or poor vascularization of the nerve. Pain may be produced by the expansion of the bone with mycetoma granuloma and grains, or it may be due to secondary bacterial infection. For unknown reasons, the tendon and the nerves usually are curiously spared until very late in the disease process; this may explain the rarity of neurological and atrophic changes even in patients with long-standing mycetoma. One of our patients presented with entrapment neuropathy, and a nerve conduction study showed evidence of focal demyelination of the median nerve at the wrist and mild acute denervation in the adductor brevis muscle, consistent with carpal tunnel syndrome, there is no obvious explanation, but it may be due to the damage caused by circulating immune complex^11^–^13^. Proximal myopathy was observed in one patient (creatinine phosphokinase was very high, muscle biopsy showed evidence of myositis, and an electromyogram study confirmed the presence of myopathy), the myopathy was most probably due to damage to the muscle with mycetoma and coexisting secondary bacterial infection, or it may be due to circulating immune complex deposition as part of a local immune response to mycetoma infection. One of our studied groups presented with spastic paraplegia due to the direct destruction of the two dorsal spinal vertebrae by mycetoma.

One of our patients is a 65 year’s Sudanese female who presented with upper and lower limb weakness; this was preceded by multiple discharging sinuses on both sides of the neck. The grains expressed through the sinus were yellow. Some of the sinuses closed transiently after discharge. First, the condition was painless; then, the patient experienced neck pain. Clinical examination showed evidence of spastic quadriplegia, while local examination of the neck showed that the skin over it becomes attached and stretched, smooth, and shiny, and there are areas of hypo and hyperpigmentation. There are subcutaneous masses with discharging sinuses; some of the old ones were healed completely. There is an area of local hyperhidrosis confined only to the site of the lesion and the skin around it. A cervical X-ray showed evidence of soft tissue swelling and destruction of cervical vertebrae C4 and C5. A cervical MRI showed extensive neck pyomyositis, epidural extension, and cord compression. The primary symptoms of a tumor, sinuses, and grains flecked discharge provided enough information to diagnose mycetoma although the species of fungi at the root of infection is identified by staining the discharge grains and inspecting them with the microscope.

Like what was mentioned in the literature mycetoma commonly affects the foot and rarely affects the head^6^,^14^. One of our patients is a 45 years Sudanese male working as a farmer who presented with progressive left-sided weakness in both upper and lower limbs; this was associated with severe headache, nausea, and vomiting. During the disease, he experienced three attacks of generalized convulsion. He claimed that he had a history of discharge containing yellow grains from a sinus in the temporal region of the head; also, he mentioned that he has no history of local trauma at the mycetoma site. The patient has had the disease for 5 years. On clinical examination, he was unwell, not pale, jaundiced, or cyanosed. Systemic examination revealed no abnormality; the abnormalities were confined to the central nervous system, where he has left-sided hemiparesis. Examination of the head showed both active and healed sinuses, yellow grains discharged from the sinuses were noted, and local hyperhidrosis around the mycetoma lesions was detected. Also he has cervical lymphadenopathy, and the regional lymph nodes were tender and attached to the skin; brain MRI examination showed intracranial extension of the disease. Although bloodstream spread in mycetoma is rare, it was reported that in such a situation, the skin and subcutaneous tissue were normal^15^,^16^. Spread along the lymphatics to the regional lymph nodes can occur, especially with actinomycetoma. Intracranial mycetoma is associated with serious complications and poor outcomes^8^,^9^.

## Conclusion

Although it is rare, clinicians should highly suspect neurological involvement in mycetoma patients. Mycetoma infection (whether bacterial or fungal) can cause peripheral or central nervous system damage. At the level of formation of a papule and discharging sinuses which can lead to entrapment neuropathy, muscle involvement by the mycetoma itself or coexist bacterial infection, direct destruction of the bone, which can cause nerve damage or cord compression. A rare manifestation is due to the spread of infection from the skull to the brain, causing hemiplegia and convulsion.

## Ethical approval

Written ethical approval was obtained from Gadarif, University Faculty of Medicine and Health Sciences, Medical Research Ethics Committee.

## Patient consent

Written informed consent was obtained from the patient for the publication of this case report and accompanying images. A copy of the written consent is available for review by the Editor-in-Chief of this journal on request.

## Sources of funding

The authors received no financial support for this research and/or authorship of this article.

## Author contribution

E.A.A. and K.A.H.M.A.: conceptualization, data curation, methodology, and writing original draft; R.T.A., A.M.A., and M.A.I.: data curation, investigation, and validation; A.S., E.O.B., A.H., and M.M.F.E.: methodology, investigation, writing, and revision of the original draft.

## Conflicts of interest disclosure

The authors declared no potential conflicts of interest.

## Research registration unique identifying number (UIN)

1. Name of the registry: NA.
2. Unique identifying number or registration ID: NA.
3. Hyperlink to your specific registration (must be publicly accessible and will be checked): NA.

## Guarantor

Mohammed Mahmmoud Fadelallah Eljack, Teaching assistant, Faculty of Medicine and Health Sciences, University of Bakht Alruda, Ad Duwaym, Sudan.

## Provenance and peer review

Not commissioned, externally peer-reviewed.

## Data availability

The materials datasets used and/or analyzed during this study are available from the corresponding author upon reasonable request.
